# Skin Biopsy and Quantitative Sudomotor Axon Reflex Testing in Patients With Postural Orthostatic Tachycardia Syndrome

**DOI:** 10.7759/cureus.31021

**Published:** 2022-11-02

**Authors:** Ryan Zhang, Ken Mayuga, Robert Shields, Christopher Cantrell, Robert Wilson

**Affiliations:** 1 Internal Medicine, Northwell Health, Long Island, USA; 2 Cardiology, Cleveland Clinic, Cleveland, USA; 3 Neurology, Cleveland Clinic Foundation, Cleveland, USA; 4 Cleveland Clinic Lerner College of Medicine, Cleveland Clinic Foundation, Cleveland, USA; 5 Neurology, Cleveland Clinic, Cleveland, USA

**Keywords:** autoimmune pots, hyperadrenergic pots, autonomic testing, hyperadrenergic orthostatic hypotension, orthostatic syndrome, skin nerve biopsy, qsart, small fiber neuropathy, neuropathic pots, pots

## Abstract

Purpose: No formal diagnostic criteria exist for the neuropathic subtype of postural orthostatic tachycardia syndrome (POTS). Skin biopsy and quantitative sudomotor axon reflex testing (QSORT) are preferred methods of assessment for autonomic small fiber neuropathy (SFN). This study characterizes the utility of these testing methods at a tertiary center and identifies clinical features associated with abnormal testing.

Methods: Medical records of 2658 patients undergoing tilt table testing at a single institution between June 2018 and December 2020 were reviewed. Patients with postural orthostatic tachycardia syndrome were included for analysis of intraepidermal nerve fiber density (IENFD), sweat output, comorbidities, symptoms, measures of cardiovascular autonomic function, and serum antibody levels.

Results: 356 patients (90% female, mean age 31 ± 10) met the diagnostic criteria for postural orthostatic tachycardia syndrome. Of 211 patients who underwent quantitative sudomotor axon reflex testing, 70 (33%) demonstrated reduced sweat output. These patients were more likely to demonstrate sympathetic impairment during the Valsalva maneuver. Of 80 patients who underwent skin biopsies, 19 (24%) demonstrated reduced intraepidermal nerve fiber density. These patients tended to be older and have reduced heart rate variability during deep breathing. Neither test was associated with specific serum antibodies, symptoms, or comorbidities, though there was a trend toward higher rates of comorbid autoimmune disease in patients with abnormal testing.

Conclusion: A subset of patients with postural orthostatic tachycardia syndrome have evidence of small fiber neuropathy. These patients tend to have impaired cardiovascular autonomic function but are otherwise similar to patients with no evidence of small fiber neuropathy.

## Introduction

Postural orthostatic tachycardia syndrome (POTS) is characterized by excessive tachycardia upon standing and symptoms of chronic orthostatic intolerance [1]. It is among the most common syndromes of autonomic dysfunction in the United States, with as many as 500,000 individuals estimated to be affected, primarily women between the ages of 13 and 50 [2]. Despite its prevalence, POTS remains poorly understood. Multiple subtypes of POTS have been proposed, including a neuropathic subtype estimated to be present in 50% of POTS patients [3]. In these patients, POTS is hypothesized to be a result of partial sympathetic denervation, which leads to inadequate vasoconstriction in the legs upon standing upright, provoking compensatory tachycardia [4]. This denervation will imply a mechanism of small fiber neuropathy (SFN). Evidence for this hypothesis includes findings of decreased norepinephrine spillover in the legs compared to the arms [5] and impaired sweat production on sudomotor testing [6-8]. In addition, some patients with neuropathic POTS demonstrate signs and symptoms of sensory SFN, including numbness, tingling, and burning [9]; sensory deficits [10]; and reduced intraepidermal nerve fiber density (IENFD) on skin biopsy [8,10,11].

No formal diagnostic criteria for neuropathic POTS exist [12]. In clinical practice, patients with suspected neuropathic POTS may undergo a variety of testing for SFN, including skin biopsy and quantitative sudomotor axon reflex testing (QSART). In the previous series, 38-45% of patients biopsied had reduced IENFD [8,10], and 33-56% of patients had reduced sweat output [6,8]. Ganglionic acetylcholine receptor (gAChR) antibodies have also been suggested as a marker of neuropathic POTS and have previously been reported to be present in up to 14-29% of patients tested [3,13]. In addition, patients with likely neuropathic POTS have been shown to demonstrate adrenergic impairment during the Valsalva maneuver, which was correlated with reduced muscle sympathetic nerve activity [14,15].

The aim of this retrospective analysis is to characterize testing for neuropathic POTS at a tertiary medical center between 2018 and 2020. The distinction between neuropathic and non-neuropathic subtypes of POTS remains unclear, and there appears to be considerable overlap between subtypes in actual clinical practice [16]. A better understanding of the utility of current testing is needed in order to identify prognostic factors and develop targeted treatment regimens. Could testing for SFN be helpful to have a better understanding of the occurrence and association of neuropathic POTS? These insights can lead to a better understanding of the etiology and treatment of POTS.

## Materials and methods

This study was approved by the institutional review board of the Cleveland Clinic, IRB 20-240. The electronic medical records of all patients aged 18 or older who underwent tilt table testing for any reason at the Cleveland Clinic between June 2018 and December 2020 were retrospectively analyzed (n = 2658). Inclusion in the cohort was contingent on meeting the following diagnostic criteria for adult POTS [16]: sustained elevation in heart rate of at least 30 beats per minute within the first 10 minutes of upright tilt [17]; absence of sustained orthostatic hypotension (a decrease in systolic blood pressure of at least 20 mmHg or a decrease in diastolic blood pressure of at least 10 mmHg) between 3 and 10 minutes of upright tilt; and symptoms of orthostatic intolerance for at least three months by the time of the initial visit (Table 1).

**Table 1:** Symptoms of orthostatic intolerance in postural orthostatic tachycardia syndrome

Lightheadedness/dizziness/presyncope
Syncope
Palpitations
Changes in sweating
Neck pain
Chest pain
Shortness of breath

Patients with a documented history of known, non-autoimmune causes of SFN-diabetic neuropathy, alcohol-related neuropathy, exposure to toxins or neurotoxic medications, and HIV neuropathy were excluded.

Additional data were collected from the electronic medical records of patients included in the final cohort for analysis (n = 356), including demographic information, medical comorbidities at the time of the initial visit, and reported symptoms at the time of the initial visit. Relevant testing results were collected when available. These included IENFD from skin biopsy, sweat output from QSART, heart rate variability and blood pressure response measures from cardiovascular autonomic function testing, and serum antibody studies.

QSART

Testing was performed using standard methods with a Q-Sweat unit (WR Medical Electronics, Maplewood, MN) [18]. Patients were tested at four sites unilaterally: the proximal foot, distal leg, proximal leg, and forearm. A 10% acetylcholine gel was iontophoresed into the skin for five minutes at each of these sites. Sweat responses were recorded for an additional five minutes. At each site, sweat output ≤5th percentile compared to established normative values for males and females was considered abnormal [19]. Patients with reduced sweat output in at least one of the four sites were considered to have an abnormal test [20,21]. Patients were asked to hold medications that might affect test reliability for five half-lives prior to testing, when permissible. 

Skin biopsy

Three-millimeter punch biopsies were obtained from patients at three sites unilaterally: distal leg, distal thigh, and proximal thigh. Skin biopsy specimens were fixed in Zamboni’s fixative, cryo-protected in 20% glycerol, and sectioned at 50 µm. Immunostaining was performed using the monoclonal mouse anti-human protein gene product 9.5 antibodies, with the immunostaining signals developed using the SG chromogen kit (Vector Laboratories, Burlingame, CA). Intraepidermal nerve fibers were counted using established counting rules to determine IENFD [22]. At each site, IENFD ≤5th percentile compared to the general population was considered abnormal [22].

Cardiovascular autonomic function tests

The cardiovagal function was assessed by the mean heart rate range (MHRR) and the expiratory-inspiratory (E:I) ratio during deep breathing. Patients were asked to take a series of successive deep breaths at a rate of approximately six breaths per minute. MHRR is calculated as the mean difference between the maximum HR during inspiration and the minimum HR during expiration [23]. The E:I ratio is calculated as the ratio of the longest R-R interval during expiration to the shortest R-R interval during inspiration [23].

Patients were instructed to perform the Valsalva maneuver for approximately 15 seconds. The Valsalva ratio, an additional measure of cardiovagal function, is calculated as the ratio of the maximum HR generated by the Valsalva maneuver to the minimum HR occurring within 30 seconds [21]. The adrenergic function was assessed by the blood pressure response to phases II and IV of the Valsalva maneuver [21]. The reviewing provider performed a visual assessment of the waveform to determine whether the phase II late and/or the phase IV response were attenuated.

Statistical analyses

Study data were collected and managed using RedCap electronic data capture tools hosted at the Cleveland Clinic Foundation [24]. Statistical analyses were performed using JMP® Pro, version 15.1.0 (SAS Institute, Inc., Cary, NC). Descriptive data are presented as mean ± SD for most continuous variables and counts and percentages for categorical variables. The median (IQR) is presented for non-normally distributed variables. Univariable analyses for continuous variables were conducted using one-way ANOVA. Univariable analyses for categorical variables were conducted using Fisher’s exact tests. For all tests of association, a standard significance threshold of p<0.05 was used.

## Results

Demographics

Among the 2658 patients who underwent tilt table testing, 356 patients met the criteria for POTS. These patients were predominantly female (90%) and white (92%), with a mean age of 31 (±10) years. The median reported duration of symptoms prior to a POTS diagnosis was three years. Of these patients, 55% were initially evaluated by a cardiologist, while 45% were initially evaluated by a neurologist.

SFN testing

Of the 356 patients with POTS, 59% underwent QSART and 22% underwent skin biopsy; 14% of patients underwent both tests. Among 51 patients who underwent both QSART and skin biopsy, there was poor agreement between the test results (Table 2). For these patients, the median time between QSART and a skin biopsy was four weeks. Males had higher rates of both reduced IENFD (16% to 7%) and reduced sweat output (32% to 27%) than females, although these differences were not statistically significant by p-value.

**Table 2:** Patients who underwent both skin biopsy and QSART (n= 51; QSART): quantitative sudomotor axon reflex testing

	Normal QSART	Abnormal QSART
Normal biopsy	18 (35%)	22 (43%)
Abnormal biopsy	6 (12%)	5 (10%)

In the overall cohort, 77 patients were tested for serum gAChR antibodies. Zero were found to have elevated antibody levels. Thirty patients were tested for serum voltage-gated potassium channel (VGKC) antibodies. Of these, only one was found to have elevated antibody levels; this patient was also found to have reduced sweat output on QSART.

Features associated with reduced IENFD

Of the 80 patients who underwent skin biopsy, 19 (24%) demonstrated reduced IENFD at least in one site. Length-dependent patterns of IENFD reduction were present in 13/19 (68%) patients with an abnormal skin biopsy. Non-length-dependent patterns were seen in 6/19 (32%).

Patients with reduced IENFD on skin biopsy were, on average, older than patients with normal IENFD (p = 0.001; Table 3). They also trended toward higher rates of comorbid autoimmune diseases, although this was not statistically significant (p = 0.10; Table 3). There were no significant differences in the frequency of reported symptoms between patients with reduced IENFD and patients with normal IENFD.

**Table 3:** Comparison of measurements of cardiovagal and adrenergic function between patients with normal IENFD on skin biopsy and patients with reduced IENFD on skin biopsy. IENFD: intraepidermal nerve fiber density.

	Normal IENFD (n = 55)	Abnormal IENFD (n = 16)	p-value
Cardiovagal function			
Deep breathing MHRR	21.6 ± 9.8	13.6 ± 5.0	0.003
Deep breathing E:I ratio	1.41 ± 0.21	1.23 ± 0.07	0.001
Valsalva ratio	2.09 ± 0.46	1.89 ± 0.42	0.13
Adrenergic function			
Abnormal BP response phase II	5%	13%	0.29
Abnormal BP response phase IV	2%	7%	0.39

Reduced IENFD was significantly associated with impaired cardiovagal function, as demonstrated by decreased MHRR (p = 0.003) and E:I ratio (o = 0.001) during deep breathing (Table 4). There was no significant association between a reduction in IENFD and attenuation of the phase II blood pressure response during the Valsalva maneuver (Table 4).

**Table 4:** Comparison of demographics and relevant comorbidities between patients with normal IENFD on skin biopsy and patients with reduced IENFD on skin biopsy. IEFND: intraepidermal nerve fiber density.

	Normal IENFD (n = 61)	Abnormal IENFD (n = 19)	p-value
Age (years)	32 ± 9	41 ± 13	0.001
Percent female	90%	84%	0.44
%White	87%	95%	0.058
Symptom duration (years)	5 (4, 6)	7 (3, 10)	0.20
Comorbidities			
Autoimmune disease (celiac, Hashimoto’s, RA, Sjogren’s)	16%	37%	0.10
Chiari	5%	2%	0.42
Ehlers-Danlos/Jjint hypermobility	31%	11%	0.13
Fibromyalgia	34%	16%	0.16
Migraine	61%	53%	0.60
Raynaud’s	23%	16%	0.75

Features associated with reduced sweat output

Of the 211 patients who underwent QSART, 70 (33%) demonstrated reduced sweat output in at least one of the four sites tested. Length-dependent patterns of sweat reduction were present in 31/70 (44%) patients with abnormal QSART, with the remaining 39/70 (56%) being non-length-dependent. Length-dependent patterns included foot only; foot and forearm; foot and distal leg; foot, distal leg, and forearm; or foot, distal leg, and proximal leg. Non-length-dependent patterns included all other patterns of sweat reduction, including global reduction.

Patients with reduced sweat output did not differ significantly from patients with normal sweat output in terms of age, frequency of reported symptoms, or medical comorbidities, although there was a trend toward elevated rates of documented autoimmune conditions among patients with reduced sweat output (p = 0.10; Table 5).

**Table 5:** Comparison of demographics and relevant comorbidities between patients with normal sweat output on QSART and patients with reduced sweat output on QSART. QSART: quantitative sudomotor axon reflex testing.

	Normal sweat output (n = 141)	Abnormal sweat output (n = 70)	p-value
Age (years)	31 ± 10	33 ± 12	0.16
Percent female	95%	90%	0.24
%White	93%	90%	0.71
Symptom duration (years)	5 (4, 6)	5 (4, 6)	0.69
Comorbidities			
Autoimmune disease (celiac, Hashimoto’s, RA, Sjogren’s)	11%	20%	0.10
Chiari	5%	0%	0.10
Ehlers-Danlos/Joint hypermobility	18%	20%	0.85
Fibromyalgia	22%	20%	0.86
Migraine	52%	50%	0.88
Raynaud’s	14%	13%	1

Compared to patients with normal sweat output on QSART, patients with reduced sweat output were more likely to demonstrate impairments in cardiovascular autonomic function testing. Patients with reduced sweat output had a lower mean Valsalva ratio (p = 0.001; Table 6). In addition, these patients demonstrated higher rates of abnormally attenuated blood pressure response to phase II of the Valsalva maneuver (p = 0.002; Table 6).

**Table 6:** Comparison of measurements of cardiovagal and adrenergic function between patients with normal sweat output on QSART and patients with reduced sweat output on QSART. QSART: quantitative sudomotor axon reflex testing.

	Normal sweat output (n = 135)	Abnormal sweat output (n = 64)	p-value
Cardiovagal function			
Deep breathing MHRR	22.4 ± 8.6	20.3 ± 8.9	0.11
Deep breathing E:I ratio	1.42 ± 0.20	1.38 ± 0.22	0.18
Valsalva ratio	2.11 ± 0.48	1.87 ± 0.40	0.001
Adrenergic function			
Abnormal BP response phase II	1%	11%	0.002
Abnormal BP response phase IV	5%	6%	0.75

## Discussion

In the absence of formal criteria for neuropathic POTS, skin biopsy and QSART are routinely used to assess for associated SFN in patients with POTS. In this cohort, one of the largest POTS cohorts studied, we identified abnormal testing in 35% of patients who underwent at least one skin biopsy and/or QSART. These patients with evidence of autonomic SFN are likely to have a neuropathic subtype of POTS, though evidence of one subtype does not rule out the presence of other subtypes, given the heterogeneity of the disorder. As has been seen in previous studies, skin biopsy and QSART results were not well correlated among the 51 patients in this cohort who underwent both tests. More than half had opposing results, with either reduced sweat output and normal IENFD or normal sweat output and reduced IENFD. This may suggest that biopsy and QSART identify distinct subsets of patients with neuropathic POTS - for example, patients with reduced sweat output may be more likely to have selective involvement of autonomic fibers and less likely to demonstrate signs and symptoms of sensory SFN. However, it is also possible that this finding reflects an overall lack of sensitivity to these tests in a condition that is thought to typically cause only mild neuropathy.

Previous studies have shown that IENFD and cardiovagal function are correlated in patients with POTS, concluding that neuropathic POTS is associated with reduced heart rate variability compared to non-neuropathic POTS [8]. We found that evidence of SFN on biopsy and QSART was associated with parasympathetic impairment, as indicated by decreased measures of heart rate variability during Valsalva and deep breathing.

In healthy individuals, phase II of the Valsalva maneuver is characterized by an initial fall in blood pressure due to a decrease in preload and stroke volume, followed by a brief rise in blood pressure mediated by sympathetic vasoconstriction [21]. Attenuation of the late phase II response indicates sympathetic impairment. In this cohort, a reduced blood pressure response to phase II of the Valsalva maneuver was associated with reduced sweat output on QSART. Out of 10 patients who demonstrated this response, 9 had an abnormal QSART, an abnormal skin biopsy, or both. This finding supports the view of neuropathic POTS as resulting from peripheral sympathetic denervation [15]. Given that a blunted phase II response was present in a small minority of patients tested, it is likely that this represents a specific but not sensitive marker for neuropathic POTS [6,8]. These patients may have relatively severe neuropathy affecting distal as well as proximal sympathetic fibers.

Phase IV of the Valsalva maneuver is normally characterized by a transient overshoot in blood pressure caused by venous return being restored while cardiac output remains elevated and the arterial bed remains vasoconstricted [25]. A previous study identified increased phase IV overshoot in non-neuropathic patients, possibly due to a hyperadrenergic state [8]. In contrast, another study found no significant differences in phase IV response between likely neuropathic POTS patients and healthy controls [14]. In this cohort, no difference was noted in blood pressure response to phase IV between neuropathic and non-neuropathic patients.

Some cases of neuropathic POTS are thought to arise from immune-mediated neuropathy. In this cohort, there was a trend toward higher rates of comorbid autoimmune disease among patients with evidence of SFN compared to patients without, though the difference was not statistically significant. Patients with systemic autoimmune diseases may be at slightly increased risk for developing a neuropathic POTS phenotype - for instance, Sjogren’s syndrome is diagnosed at higher rates among POTS patients who have reduced IENFD [26].

Specific antibodies that have been proposed as potential markers for neuropathic POTS include gAChR antibodies and VGKC antibodies. Nicotinic AChRs mediate synaptic transmission in the autonomic ganglia. High levels of gAChR antibodies have been identified as a defining feature of autoimmune autonomic ganglionopathy (AAG) [27], a rare cause of diffuse autonomic failure. Low levels of gAChR antibodies have been identified in up to 14-29% of POTS patients in previous series [3,13]. At our tertiary medical center, gAChR antibodies were tested in 77 patients, representing approximately one fifth of the patients studied. All POTS patients tested were found to be seronegative. In a previous study conducted at the same institution, 95 out of 6032 patients tested were identified as seropositive for gAChR antibodies, three of whom carried a diagnosis of POTS [28]. These three patients all had low levels of gAChR antibodies (<0.12 nmol/L). The lack of association between gAChR antibodies and POTS was also seen in a study of POTS patients in the community, which detected seropositivity in only 7% of patients, a figure comparable to that found in healthy controls [29]. Taken together, these findings suggest that gAChR antibodies are rarely elevated in POTS patients and are unlikely to be a relevant marker for distinguishing between subtypes of POTS.

VGKC antibodies have been detected in Morvan syndrome, which is associated with a constellation of symptoms that include peripheral neuropathy and autonomic dysfunction, leading to speculation that they may underlie some cases of POTS [4,30]. In this cohort, VGKC antibodies were tested in 30 patients, representing approximately one-tenth of the patients studied. Only one patient was found to have levels above the normal range; this patient was in the indeterminate range (32-87 pmol/L). As with gAChR antibodies, VGKC antibodies are unlikely to be prevalent enough in POTS patients to help distinguish between different subtypes.

Limitations

This study has a number of limitations. The findings reported here reflect a single large tertiary center in the U.S. and thus may not be generalizable to other types of practice. In addition, due to the retrospective nature of data collection, patients included in the cohort did not receive a standardized battery of tests. Rather, they were tested based on clinical judgment, which will vary between providers or across departments. Another limitation of the retrospective design is the reliance on provider documentation for information on patient symptoms and histories. In future studies, the prospective application of a standardized questionnaire would help to overcome this limitation. Additionally, many patients may demonstrate features of both neuropathic and non-neuropathic subtypes, indicating that these groupings are overlapping rather than distinct. It is possible that some patients who had abnormal SFN testing also had concomitant hyperadrenergic or hypovolemic pathophysiology.

## Conclusions

This retrospective study aims to characterize testing for SFN in patients with POTS seen at a tertiary medical center. Skin biopsy and QSART are commonly used tests for neuropathic POTS that are not correlated with each other and may not be typically length-dependent. Evidence of SFN on either of these tests is associated with impaired cardiovascular autonomic function, and reduced IENFD on biopsy in particular is more likely to be seen in older POTS patients. However, specific symptoms, comorbidities, and serum antibodies are not significantly associated with biopsy and QSART results, suggesting that POTS patients with SFN appear clinically similar to POTS patients with no evidence of SFN. These results further our understanding of the utility of current testing for neuropathic POTS, a question that is relevant for the future development of consensus criteria and targeted therapies. There is an occurrence of small fiber neuropathy that occurs in POTS. Future research in more prospective studies may help delineate and enable a further refined understanding of the relationship between small-fiber neuropathy and POTS.

## References

[1] Schondorf R, Low PA (1993). Idiopathic postural orthostatic tachycardia syndrome: an attenuated form of acute pandysautonomia?. Neurology.

[2] Garland EM, Celedonio JE, Raj SR (2015). Postural tachycardia syndrome: beyond orthostatic intolerance. Curr Neurol Neurosci Rep.

[3] Thieben MJ, Sandroni P, Sletten DM (2007). Postural orthostatic tachycardia syndrome: The Mayo Clinic experience. Mayo Clinic Proc.

[4] Benarroch EE (2012). Postural tachycardia syndrome: a heterogeneous and multifactorial disorder. Mayo Clin Proc.

[5] Jacob G, Costa F, Shannon JR (2000). The neuropathic postural tachycardia syndrome. N Engl J Med.

[6] Peltier AC, Garland E, Raj SR (2010). Distal sudomotor findings in postural tachycardia syndrome. Clin Auton Res.

[7] Al-Shekhlee A, Lindenberg JR, Hachwi RN, Chelimsky TC (2005). The value of autonomic testing in postural tachycardia syndrome. Clin Auton Res.

[8] Gibbons CH, Bonyhay I, Benson A, Wang N, Freeman R (2013). Structural and functional small fiber abnormalities in the neuropathic postural tachycardia syndrome. PLoS One.

[9] Shaw BH, Stiles LE, Bourne K (2019). The face of postural tachycardia syndrome - insights from a large cross-sectional online community-based survey. J Intern Med.

[10] Billig SC, Schauermann JC, Rolke R, Katona I, Schulz JB, Maier A (2020). Quantitative sensory testing predicts histological small fiber neuropathy in postural tachycardia syndrome. Neurol Clin Pract.

[11] Haensch CA, Tosch M, Katona I, Weis J, Isenmann S (2014). Small-fiber neuropathy with cardiac denervation in postural tachycardia syndrome. Muscle Nerve.

[12] Goodman BP (2018). Evaluation of postural tachycardia syndrome (POTS). Auton Neurosci.

[13] Watari M, Nakane S, Mukaino A (2018). Autoimmune postural orthostatic tachycardia syndrome. Ann Clin Transl Neurol.

[14] Jacob G, Diedrich L, Sato K (2019). Vagal and sympathetic function in neuropathic postural tachycardia syndrome. Hypertension.

[15] Sandroni P, Novak V, Opfer-Gehrking TL, Huck CA, Low PA (2000). Mechanisms of blood pressure alterations in response to the Valsalva maneuver in postural tachycardia syndrome. Clin Auton Res.

[16] Arnold AC, Ng J, Raj SR (2018). Postural tachycardia syndrome - diagnosis, physiology, and prognosis. Auton Neurosci.

[17] Mayuga KA, Butters KB, Fouad-Tarazi F (2008). Early versus late postural tachycardia: a re-evaluation of a syndrome. Clin Auton Res.

[18] Low PA, Caskey PE, Tuck RR, Fealey RD, Dyck PJ (1983). Quantitative sudomotor axon reflex test in normal and neuropathic subjects. Ann Neurol.

[19] Sletten D, Grandinetti A, Weigand S, Gehrking T, Gehrking J, Low P, Singer W (2015). Normative values for sudomotor axon reflex testing using QSWEATTM (P1.282). Neurology.

[20] Fabry V, Gerdelat A, Acket B (2020). Which method for diagnosing small fiber neuropathy?. Front Neurol.

[21] Low PA, Tomalia VA, Park KJ (2013). Autonomic function tests: some clinical applications. J Clin Neurol.

[22] Tavee JO, Polston D, Zhou L, Shields RW, Butler RS, Levin KH (2014). Sural sensory nerve action potential, epidermal nerve fiber density, and quantitative sudomotor axon reflex in the healthy elderly. Muscle Nerve.

[23] Shields RW Jr (2009). Heart rate variability with deep breathing as a clinical test of cardiovagal function. Cleve Clin J Med.

[24] Harris PA, Taylor R, Thielke R, Payne J, Gonzalez N, Conde JG (2009). Research electronic data capture (REDCap): a metadata-driven methodology and workflow process for providing translational research informatics support. J Biomed Inform.

[25] Wada N, Singer W, Gehrking TL, Sletten DM, Schmelzer JD, Low PA (2014). Comparison of baroreflex sensitivity with a fall and rise in blood pressure induced by the Valsalva manoeuvre. Clin Sci (Lond).

[26] Pasadyn SR, Warren CB, Wilson RG (2020). Utility of salivary gland biopsy in diagnosing Sjogren's syndrome in a POTS patient population. Auton Neurosci.

[27] Vernino S, Stiles LE (2018). Autoimmunity in postural orthostatic tachycardia syndrome: current understanding. Auton Neurosci.

[28] Li Y, Jammoul A, Mente K, Li J, Shields RW, Vernino S, Rae-Grant A (2015). Clinical experience of seropositive ganglionic acetylcholine receptor antibody in a tertiary neurology referral center. Muscle Nerve.

[29] Bryarly M, Raj SR, Phillips L (2021). Ganglionic acetylcholine receptor antibodies in postural tachycardia syndrome. Neurol Clin Pract.

[30] Golden EP, Vernino S (2019). Autoimmune autonomic neuropathies and ganglionopathies: epidemiology, pathophysiology, and therapeutic advances. Clin Auton Res.

